# Reallocating 24-hour movement behaviors and its impact on mental health in preschool children: a compositional data and dose-response analysis

**DOI:** 10.1186/s13034-025-00911-7

**Published:** 2025-05-09

**Authors:** Tian Wang, Junyu Wang, Xuelin Lu, Xuhui Chen, Lanzhi Chen, Yixin Liang, DongQing Yang, Yanmei Shi, Rui Li, Yong Yang, Beibei Luo, Jie Zhuang

**Affiliations:** 1 School of Exercise and Health, Shanghai University of Sport, 399 Changhai Rd, Yangpu District, Shanghai, 200438 China; 2 Officers College of PAP, Chengdu, China; 3 School of Physical Education, Shanghai University of Sport, Shanghai, China; 4 Experimental Kindergarten Attached To Shanghai University, Shanghai, China; 5 School of Physical Education and Sport, Chaohu University, Hefei, China; 6 Shanghai Student Physical Fitness and Health Research Center, Shanghai, China

**Keywords:** Preschool children, Mental health, 24-hour movement behaviors, Compositional data analysis, Isotemporal substitution, Dose-response relationship

## Abstract

**Background:**

Mental health issues in preschool children are a significant public health concern with long-term developmental implications. Understanding how reallocations of time among different 24-hour movement behaviors-moderate-to-vigorous physical activity (MVPA), light physical activity (LPA), sedentary behavior (SED), and sleep (SLP)-affect various dimensions of mental health is essential for designing effective interventions.

**Methods:**

In this cross-sectional study, 828 Chinese preschool children aged 3 to 6 years were assessed. Physical activity and sleep were objectively measured using accelerometers to capture MVPA, LPA, SED, and SLP. Mental health was evaluated using the Strengths and Difficulties Questionnaire (SDQ), assessing Total Difficulties, Internalizing Problems, Externalizing Problems, and Prosocial Behavior. Compositional data analysis was conducted using isometric log-ratio (ilr) transformation, followed by multivariate linear regression to assess associations. Additionally, isotemporal substitution modeling and dose-response analysis were applied to examine the effects of reallocating time between behaviors on mental health outcomes, adjusting for age, gender, BMI, parental education, and parental attitudes toward physical activity.

**Results:**

Increased MVPA was significantly associated with lower Total Difficulties (ß_MVPA_= − 1.587; *P* < 0.001) and Internalizing Problems (ß_MVPA_= − 0.663; *P* = 0.017). Increased SED was associated with higher Total Difficulties (ß_SED_= 1.512; *P* < 0.05), while increased SLP was linked to improved Externalizing Problems (ß_LPA_= − 1.792; *P* = 0.008). Reallocating as little as 1 min from LPA or SED to MVPA or SLP significantly reduced Total Difficulties and Internalizing Problems (*P* < 0.05). Conversely, replacing SED or SLP with LPA increased Externalizing Problems, particularly Conduct Problems and Hyperactivity/Inattention (*P* < 0.05). A critical threshold at 30 min was identified, beyond which the effects on mental health outcomes became more pronounced.

**Conclusions:**

Reallocating time from LPA or SED to MVPA or SLP significantly improves Total Difficulties and Internalizing Problems in preschool children, even with brief substitutions. However, increasing LPA at the expense of SED or SLP exacerbates Externalizing Problems, especially Conduct Problems and Hyperactivity/Inattention. Interventions should promote MVPA and adequate sleep while considering the critical 30-minute threshold where effects become more pronounced to enhance emotional and behavioral well-being.

**Supplementary Information:**

The online version contains supplementary material available at 10.1186/s13034-025-00911-7.

## Background

Mental health issues among children have emerged as a significant global public health concern, with approximately 20% of children and adolescents worldwide experiencing mental health disorders such as depression, anxiety, and behavioral problems [1]. A global meta-analysis reported a pooled prevalence of 13.4% for any mental disorder in children and adolescents, with variations across regions and age groups [2]. In China, around 17% of children are affected, highlighting the pervasive nature of this issue [3]. Specifically, studies have shown that more than 25% of Chinese preschool children exhibit emotional or behavioral difficulties, such as hyperactivity, emotional instability, and challenging behavior in social interaction [4, 5], indicating that mental health challenges emerge even before school age. These early difficulties not only diminish quality of life during childhood but also have long-term consequences on psychological development, social adaptation, and academic performance into adulthood [6]. If not addressed promptly, these issues may persist, exacerbating social and economic burdens [7, 8].

The preschool period is a critical stage for psychological development and presents a vital window for early detection and intervention. Preschool children’s mental health is complex and multifaceted, encompassing various dimensions such as emotional symptoms, conduct problems, hyperactivity/inattention, peer relationship problems, and prosocial behaviors [9]. Understanding the factors influencing these dimensions is essential for developing targeted interventions. Physical activity and sleep have been identified as significant influencers of children’s mental health. Moderate-to-vigorous physical activity (MVPA) and adequate sleep (SLP) are particularly crucial for emotional regulation and behavioral outcomes [10, 11].

In recent years, a growing body of evidence has reinforced the theoretical foundation linking 24-hour movement behaviors—including MVPA, light physical activity (LPA), sedentary behavior (SED), and sleep—to psychosocial well-being in early childhood. Notably, Carson et al. and Poitras et al. found that adherence to integrated movement guidelines was associated with better emotional regulation, reduced anxiety, and fewer behavioral problems in children under five [12, 13]. Similarly, Chaput et al. emphasized that insufficient sleep and excessive sedentary time adversely affect mood and social functioning in young children [14]. These studies formed the basis of international 24-hour movement guidelines, which adopt a holistic perspective on how the co-dependent distribution of daily activities collectively influences mental health. However, recent research emphasizes the need for a holistic view of 24 h movement behaviors to fully understand their cumulative impact on health [15]. This integrative perspective is especially relevant to preschoolers, whose daily activity is distributed across highly variable, short-duration movement episodes.

Compositional data analysis offers an effective method for examining the proportional allocation of activities within the 24-hour day, considering the interdependent nature of movement behaviors. This approach allows for isotemporal substitution analysis, where time spent on one activity is reallocated to another while keeping total time constant [16–18]. Studies utilizing this framework have demonstrated that replacing sedentary time with MVPA or sleep can improve physical and psychological outcomes in children [19, 20]. Nevertheless, most of these studies have focused on school-aged children or adolescents, with limited evidence specific to preschoolers—a group with unique developmental and behavioral characteristics. Moreover, existing research has not thoroughly analysed the relationship between the distribution of 24 h movement behaviors and different dimensions of preschool children’s mental health. This gap leaves the complex interactions between various movement behaviors and their impact on specific mental health dimensions underexplored. Furthermore, most studies have focused on larger time increments (e.g., 5 to 60 min) and have not fully explored the effects of shorter reallocations. Given that preschool children’s movement patterns are characterized by rapid fluctuations and frequent short bouts of activity [21], investigating micro-level substitutions may be more ecologically valid and informative for this age group.

Therefore, this study aims to: (1) explore the relationship between 24-hour movement behaviors and different dimensions of preschool children’s mental health using compositional data analysis; (2) analyse the impact of short-term 24-hour movement behavior substitutions on preschool children’s mental health, focusing on small time increments that reflect their natural activity patterns; and (3) investigate the dose-response relationship of time reallocations among movement behaviors on mental health outcomes. This approach is expected to contribute novel insights into how subtle changes in movement behavior distribution relate to emotional and behavioral outcomes in early childhood, thereby guiding the development of more effective, targeted intervention strategies.

## Methods

### Study design and participants

This cross-sectional study employed a combination of stratified cluster random sampling and convenience sampling to ensure the representativeness of the sample. Three urban districts were randomly selected from the 16 districts of Shanghai, China. Within each district, one kindergarten was randomly chosen, and preschool children aged 3 to 6 years from these kindergartens were recruited as the study participants. Initially, 1080 preschool children were invited to participate in the study. After obtaining written informed consent from parents or guardians, 1017 children voluntarily participated. Children with significant physical, cognitive, or psychological impairments were excluded from the study. After this screening, 994 children met the inclusion criteria. Of the participants, 861 children provided valid 24-hour movement data (86.6%), and demographic and mental health questionnaires were collected from 840 children (83.3%). After merging both datasets, 828 children were included in the final analysis, with an effective data rate of 83.3%.

The study was approved by the Institutional Review Board of Shanghai University of Sport (Ethics Approval No.: 102772023RT050). All participating children and their parents or guardians provided verbal informed consent after being fully informed about the study procedures.

### Procedure

Before data collection, all research personnel received comprehensive training. The research team, comprising postgraduate students in sports science, explained the study objectives and procedures to participants and their parents in a classroom setting. During the accelerometer data collection phase, research staff visited the kindergartens daily to ensure proper usage of the accelerometers and data accuracy. Parents were instructed on how to correctly fit and remove the devices. To assess mental health outcomes, parents completed electronic questionnaires, with real-time progress monitored through a backend system, allowing for verification and cross-checking of responses. After data collection, a systematic review was conducted to identify and resolve missing or duplicated data. A double-entry system was used for data accuracy, with two assistants independently entering data into a secure database, followed by cross-verification to ensure consistency.

All demographic, accelerometer, and questionnaire data were anonymized using unique identification codes to maintain confidentiality, and access to the database was restricted to authorized researchers to ensure data privacy and security.

### Measures

#### 24 h movement behaviors measurement

Preschool children’s 24 h movement behaviors were measured using the wGT3X-BT triaxial accelerometer (referred to as “accelerometer”). Prior to testing, a meeting was held with parents and teachers to explain the study procedures and provide instructions on wearing and removing the device. The accelerometer was initialized with a 15-second epoch, and children wore it on their right hip for seven consecutive days. Data collection began at 23:00 on the first day and continued until the device was retrieved on the eighth day.

Data were processed using Actilife 6.5 software. To ensure validity, children had to wear the accelerometer for at least three valid days, including two weekdays and one weekend day, with a minimum of 10 h per day, excluding water-based activities such as bathing or swimming [22]. Physical activity intensity and sedentary behavior (SED) were classified according to cut-points for preschool children defined by Pate et al. [23]. Specifically, SED was defined as 0–799 counts/min, light physical activity (LPA) as 800–1679 counts/min, moderate physical activity (MPA) as 1680–3367 counts/min, and vigorous physical activity (VPA) as ≥ 3368 counts/min. To improve practical interpretation, these categories can be illustrated by common daily examples: SED includes sitting still while watching television, drawing, or listening to a story; LPA involves slow walking, pretend play, or tidying up toys; and MVPA (i.e., MPA + VPA) includes running, playing tag, jumping, or dancing. Total sleep duration (SLP), including naps, was calculated using the algorithm developed by Sadeh et al. [24], validated for preschool populations by Meredith-Jones et al. [25].

The primary outcomes were moderate-to-vigorous physical activity (MVPA, calculated as MPA + VPA), LPA, SED, and SLP, all derived from the accelerometer data.

#### Mental health assessment

Children’s mental health was assessed using the Chinese version of the Strengths and Difficulties Questionnaire (SDQ), which consists of 25 items across five subscales: Emotional Symptoms, Peer Relationship Problems, Conduct Problems, Hyperactivity/Inattention, and Prosocial Behavior. Each item is rated on a 3-point scale, with parents completing the electronic questionnaire based on their child’s daily behavior. The Chinese SDQ version has been extensively validated for use in preschool-aged populations and demonstrates acceptable psychometric properties. Previous studies have reported good internal consistency (Cronbach’s alpha ranging from 0.63 to 0.78 across subscales), satisfactory test–retest reliability (intraclass correlation coefficients > 0.70), and robust construct validity, as confirmed by confirmatory factor analysis in Chinese samples aged 3 to 6 years [26–28].

Each SDQ subscale contains 5 items, with scores ranging from 0 to 10. The Total Difficulties Score (range 0–40) is calculated by summing the scores of four subscales: Emotional Symptoms, Peer Relationship Problems, Conduct Problems, and Hyperactivity/Inattention. In line with previous recommendations for general and low-risk populations [29], the study also calculated Internalizing Problems (sum of Emotional Symptoms and Peer Relationship Problems) and Externalizing Problems (sum of Conduct Problems and Hyperactivity/Inattention). Lower Total Difficulties, Internalizing Problems, and Externalizing Problems scores indicate better mental health. Prosocial Behavior is reported separately, with higher scores reflecting better social functioning.

####  Covariates

Previous studies have demonstrated that factors such as gender, age, parental education level, and parental attitudes toward physical activity can significantly influence children’s 24-hour movement behaviors [30]. To account for potential confounding effects, the following covariates were controlled for in the analysis: child’s age, gender, district of residence, BMI, parental education level, and parental attitudes toward physical activity.

Age, gender, district of residence, and parental education level were collected through a structured questionnaire. Parental attitudes toward physical activity were assessed with a dichotomous response (“Like” or “Dislike”). BMI was calculated as weight (kg) divided by height squared (m^2^). These covariates were included in the statistical models to ensure a robust analysis of the relationship between 24-hour movement behaviors and mental health outcomes.

### Statistical analysis

Statistical analyses followed the guidelines for 24-hour movement behavior composition analysis as described by Chastin et al. [16]. All analyses were conducted using R version 4.3.2 with the Compositions and robCompositions packages, and Stata 17.0 software. The specific procedures included:

#### Descriptive statistics for 24 h movement

The total 24-hour movement time was fixed at 1440 min. Mean values of each movement component (MVPA, LPA, SED, and SLP) were calculated to describe central tendencies. A log-ratio variance matrix was employed to assess the variability among these behaviors, where lower variance values indicated stronger associations between two behaviors and higher values indicated weaker associations.

#### Multivariate linear regression for 24 h movement

To address multicollinearity inherent in compositional data, isometric log-ratio (ilr) transformations were applied to the movement behavior components. These ilr-transformed components were then used as independent variables in multivariate linear regression models, with mental health outcomes as dependent variables. Covariates, including age, gender, district of residence, BMI, parental education level, and parental attitudes toward physical activity, were incorporated into the models to control for potential confounding effects. This allowed for an in-depth analysis of how different activity behaviors are associated with mental health in preschool children.

#### Isotemporal substitution analysis

Following the multivariate linear regression, isotemporal substitution analysis was performed to evaluate the effects of reallocating time between different movement behaviors on mental health outcomes [31]. The ilr-transformed data were used to predict changes in the outcome variables when a fixed amount of time (e.g., 5 min) was substituted from one activity to another, while keeping the total time spent on all activities constant.

Considering that short bouts of MVPA (< 5 min) constitute a significant portion of preschool children’s daily activities, particularly in China [21], this analysis began by reallocating time in 1-minute and 5-minute increments. The total time was fixed at 1440 min, and the reallocation of 1–5 min from one activity to another was modeled, keeping the time spent on the remaining activities unchanged. The impact of these substitutions on mental health outcomes was then calculated.

For behaviors showing significant associations with mental health in the isotemporal substitution analysis, a further dose-response analysis was conducted. Time reallocations were extended in 5-minute increments, up to 60 min, to explore the dose-response relationship between time substitutions and mental health outcomes. Both the isotemporal substitution and dose-response analyses were performed using the Compositions package in R [31, 32], with data visualization and plotting completed in Excel 2021, following established compositional data analysis methods.

## Results

### Participant characteristics

The final sample included 828 preschool children (mean age 4.9 ± 0.9 years; mean BMI 15.7 ± 1.7 kg/m²), with 449 boys (54.2%) and 379 girls (45.8%). Class levels were distributed as 14.7% junior (*n* = 122), 28.4% middle (*n* = 235), and 56.9% senior (*n* = 471). Regarding family structure, 68.8% of children were the only child. Parental education levels were 1.7% primary/junior high, 6.8% high school/technical, 70.0% bachelor’s/associate, and 21.5% master’s or higher. Parental attitudes toward physical activity varied: 23.6% of parents strongly liked it, 29.4% moderately liked it, 38.0% were neutral, and 9.0% disliked it. The mean Total Difficulties Score was 9.05 ± 3.25. The mean Internalizing Problems score was 3.99 ± 2.20, Externalizing Problems 5.68 ± 2.49, and Prosocial Behavior 6.85 ± 1.82 (see Table 1).

**Table 1:** Characteristics of participants

Variable	Percentage (%) / Mean ± SD
Age (years)	4.91 ± 0.92
BMI (kg/m²)	15.74 ± 1.71
Gender (n, %)	
Boy	449 (54.2)
Girl	379 (45.8)
District (n,%)	
Baoshan district	464 (56.0)
Jing’an district	121 (14.6)
Minhang district	243 (29.4)
Class Level (n, %)	
Junior class	122 (14.7)
Middle class	235 (28.4)
Senior class	471 (56.9)
Only Child (n, %)	
Yes	570 (68.8%)
No	258 (31.2%)
Parental education level (n, %)	
Primary/junior high school	14 (1.7%)
High school/vocational school	56 (6.8%)
Bachelor/associate degree	580 (70.0%)
Master’s degree or higher	178 (21.5%)
Parental Attitudes toward physical activity (n, %)	
Very positive	195 (23.6%)
Somewhat positive	243 (29.4%)
Neutral	315 (38.0%)
Not positive	75 (9.0%)
Mental health scores	
Total difficulties score	9.05 ± 3.25
Internalizing problems	3.99 ± 2.20
Emotional symptoms	1.93 ± 1.51
Peer relationship problems	2.06 ± 1.31
Externalizing problems	5.68 ± 2.49
Conduct problems	1.82 ± 1.06
Hyperactivity/Inattention	3.85 ± 1.94
Prosocial Behavior	6.85 ± 1.82

###  Distribution of 24 h movement

#### Descriptive analysis

Compositional mean times (and percentage of 24-hour time) were 78.87 min (5.48%) for MVPA, 112.73 min (7.83%) for LPA, 649.32 min (45.09%) for SED, and 599.08 min (41.60%) for SLP. The corresponding arithmetic means were 77.50 min (5.70%) for MVPA, 96.50 min (7.14%) for LPA, 552.50 min (40.75%) for SED, and 629.00 min (46.42%) for SLP (Table 2). The arithmetic method slightly underestimated MVPA, LPA, and SED, and overestimated SLP, highlighting the value of compositional analysis for accurate movement behavior representation.

**Table 2:** Compositional mean and arithmetic mean of 24-hour movement data

Statistic	MVPA	LPA	SED	SLP
Compositional Mean (min)	78.87	112.73	649.32	599.08
Compositional Mean (%)	5.48	7.83	45.09	41.60
Arithmetic Mean (min)	77.50	96.50	552.50	629.00
Arithmetic Mean (%)	5.70	7.14	40.75	46.42

#### Variance matrix analysis

The log-ratio variance matrix (Supplementary Table 1) showed all isometric log-ratio variances > 0, indicating interdependence of behaviors. The lowest variance was between SED and SLP, suggesting a high degree of substitution between these two behaviors. The highest variances were between MVPA vs. LPA and LPA vs. SED, indicating those behaviors are less interchangeable.

### Compositional linear regression analysis of 24 h movement and mental health

After adjusting for covariates, ilr-transformed 24-hour movement behaviors (MVPA, LPA, SED, SLP) were entered into linear regression models with SDQ subscales (Emotional Symptoms, Peer Problems, Conduct Problems, Hyperactivity/Inattention, Prosocial Behavior) and summary scores (Total Difficulties, Internalizing, Externalizing) as outcomes (Table 3). For Total Difficulties (model *P* < 0.001, R² = 0.048), MVPA was negatively associated (β = -1.587, *P* < 0.001) and SED was positively associated (β = 1.511, *P* < 0.05) with the score. LPA and SLP showed no significant associations (*P* > 0.05). For Internalizing Problems (model *P* = 0.045, R² = 0.016), MVPA had a negative association (β = -0.663, *P* = 0.017), while LPA, SED, and SLP were not significantly associated. In the Emotional Symptoms subscale, MVPA was negatively (β = -0.423, *P* = 0.026) and LPA positively (β = 0.383, *P* = 0.038) associated, whereas SED and SLP were not. No movement behavior was significantly related to Peer Problems (*P* > 0.05). For Externalizing Problems (model *P* = 0.053, R² = 0.015), SED (β = -1.325, *P* = 0.022) and SLP (β = -1.792, *P* = 0.008) were negatively associated, while MVPA and LPA were not significant. In the Conduct Problems subscale, MVPA was negatively (β = -0.300, *P* = 0.024) and LPA positively (β = 0.397, *P* = 0.002) associated. For Hyperactivity/Inattention, MVPA showed a positive association (β = 0.587, *P* = 0.016) and both SED (β = -1.150, *P* = 0.011) and SLP (β = -1.551, *P* = 0.003) showed negative associations. No significant associations were observed between any movement behavior and Prosocial Behavior (*P* > 0.05).

**Table 3:** Compositional linear regression between the proportion of 24-hour movement and mental health outcomes

Outcome	24-hour Movement	β	*P*	Model *P*-value	Model *R*²
Total difficulties Score	MVPA	-1.587	<0.001**	<0.001	0.047
	LPA	0.732	0.061		
	SED	1.511	0.042*		
	SLP	-0.986	0.252		
Internalizing problems	MVPA	-0.663	0.017*	0.045	0.016
	LPA	0.452	0.093		
	SED	0.393	0.442		
	SLP	-0.297	0.615		
Emotional symptoms	MVPA	-0.423	0.026^*^	0.006	0.022
	LPA	0.383	0.038^*^		
	SED	0.118	0.737		
	SLP	-0.512	0.206		
Peer relationship problems	MVPA	-0.240	0.145	0.024	0.018
	LPA	0.069	0.669		
	SED	0.275	0.366		
	SLP	0.215	0.542		
Externalizing problems	MVPA	0.287	0.360	0.053	0.015
	LPA	0.512	0.094		
	SED	-1.325	0.022*		
	SLP	-1.792	0.008*		
Conduct Problems	MVPA	-0.300	0.024*	0.034	0.016
	LPA	0.397	0.002*		
	SED	-0.175	0.476		
	SLP	-0.242	0.396		
Hyperactivity/Inattention	MVPA	0.587	0.016*	0.012	0.019
	LPA	0.114	0.628		
	SED	-1.150	0.011*		
	SLP	-1.551	0.003*		
Prosocial	MVPA	0.163	0.478	0.170	0.011
	LPA	-0.109	0.626		
	SED	-0.098	0.818		
	SLP	0.424	0.388		

Models were adjusted for covariates (age, gender, district of residence, BMI, parental education level, and parental attitudes toward physical activity). * indicates *P* < 0.05, ** indicates *P* < 0.01.

### Predicted changes in mental health following isotemporal substitution of 24 h movement

Isotemporal substitution models were used to estimate the impact of reallocating time between behaviors in 1-minute and 5-minute increments (Supplementary Table 2). After adjusting for covariates, the following effects were observed:

Replacing 1 min of LPA or SED with MVPA significantly reduced the Total Difficulties Score (95% CI of change for LPA: -0.033 to -0.011; for SED: -0.026 to -0.009). Similarly, replacing 1 min of LPA or SED with SLP lowered the Total Difficulties Score (LPA: -0.020 to -0.006; SED: -0.013 to -0.005). These effects were consistent for 5-minute reallocations.

For Internalizing Problems, reallocating 1 min from LPA or SED to MVPA significantly lowered the score (LPA: -0.018 to -0.002; SED: -0.013 to -0.001). Likewise, shifting 1 min from LPA or SED to SLP reduced Internalizing Problems (LPA: -0.010 to -0.001; SED: -0.005 to -0.001). Emotional Symptoms showed similar improvements with these reallocations, while Peer Problems remained unchanged.

For Externalizing Problems, replacing 1 min of SED or SLP with LPA (i.e., increasing LPA at the expense of SED or SLP) raised the score (SED: 0.001 to 0.009; SLP: 0.002 to 0.012). Specifically, for Conduct Problems, replacing 1 min of MVPA, SED, or SLP with LPA increased scores (MVPA: 0.002 to 0.010; SED: 0.001 to 0.005; SLP: 0.001 to 0.006). Additionally, reallocating 1 min of MVPA to SED increased Conduct Problems (0.001 to 0.006). For Hyperactivity/Inattention, shifting 1 min from SED or SLP to MVPA led to higher scores (SED: 0.008 to 0.061; SLP: 0.014 to 0.072). The 5-minute substitution results were consistent with those of 1-minute increments.

No significant changes in Prosocial Behavior were observed with any time reallocations (*P* > 0.05).

###  Dose-response relationship of 24 h movement substitution

**Fig. 1:**
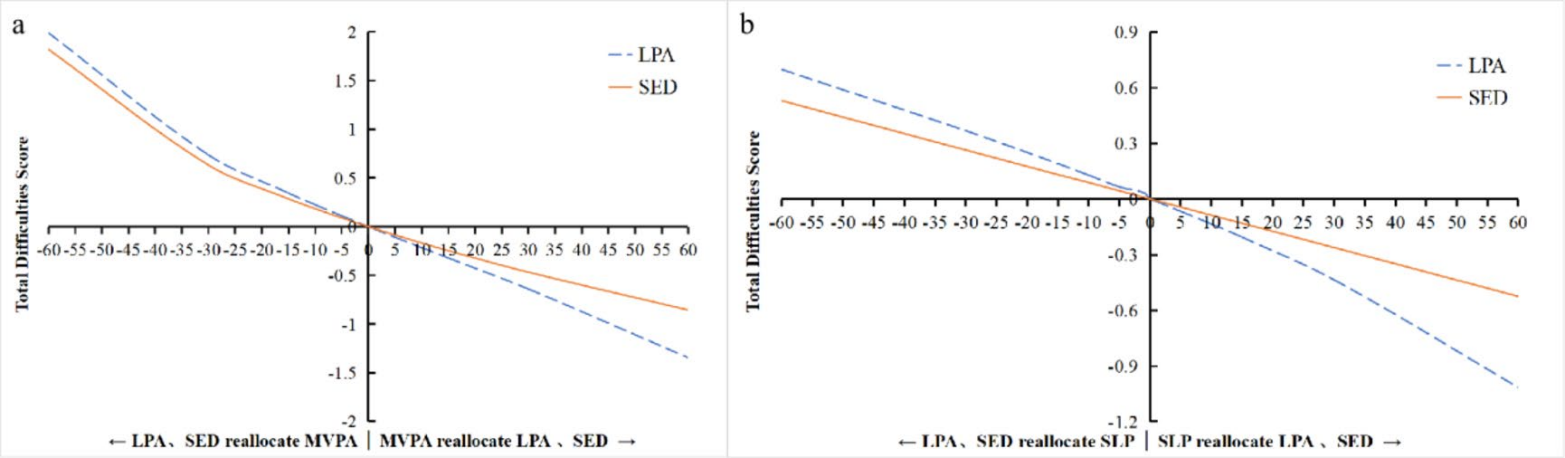
Effects of Isotemporal Substitution on Total Difficulties Score. **a** Substituting LPA and SED with MVPA. **b** Substituting LPA and SED with SLP

**Fig. 2:**
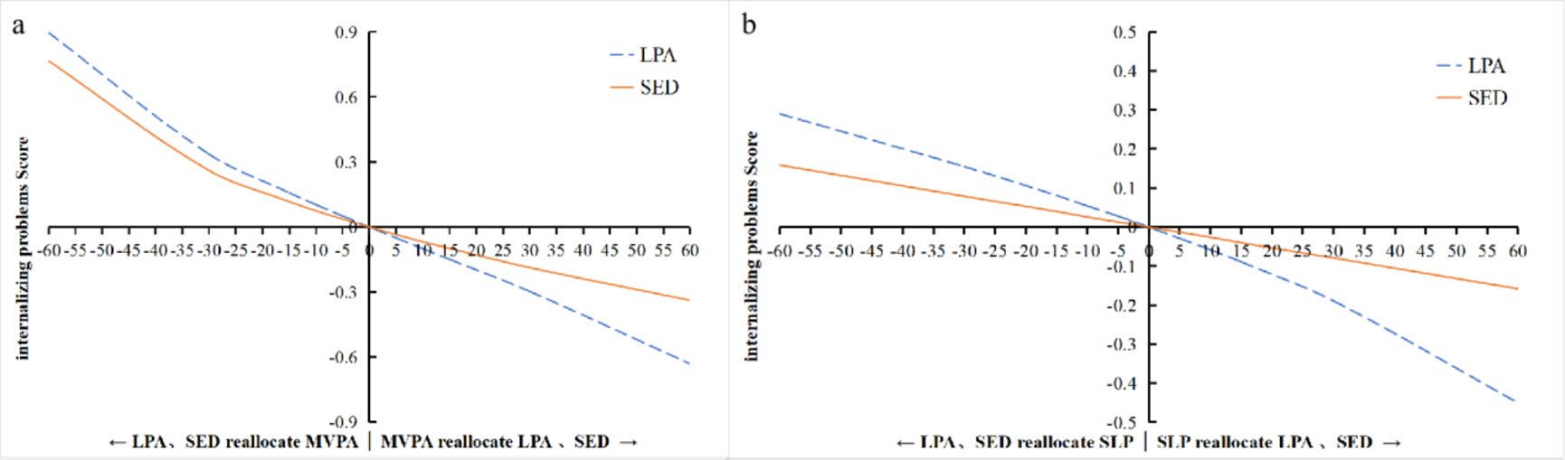
Effects of Isotemporal Substitution on Internalizing Problems. **a** Substituting LPA and SED with MVPA. **b** Substituting LPA and SED with SLP

**Fig. 3:**
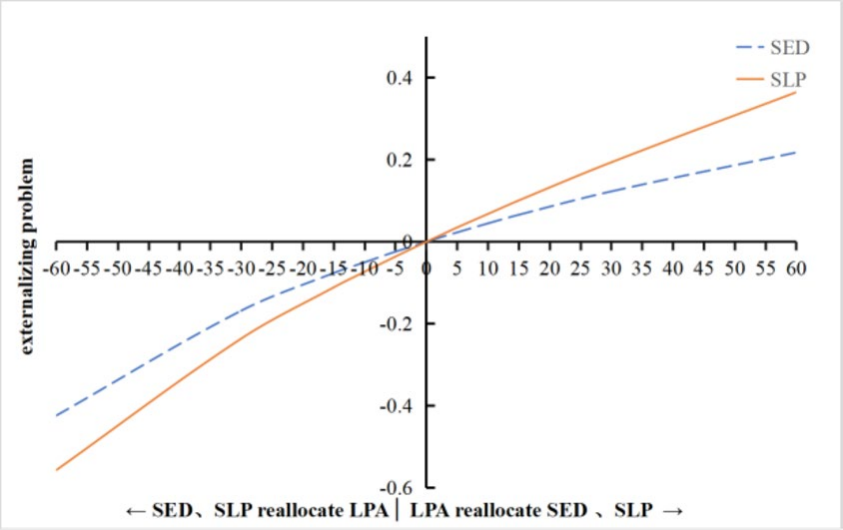
Effect of Isotemporal Substitution of SED and SLP with LPA on Externalizing Problems

To further examine the substitution effects, time reallocations were simulated from 1 up to 60 min in 5 min increments, as illustrated in Figs. 1, 2 and 3:

1. Total Difficulties (Fig. 1a and b): Increasing MVPA (replacing LPA or SED) led to a progressively lower Total Difficulties Score, with the greatest reduction when replacing LPA. Conversely, reducing MVPA (adding LPA or SED) increased the Total Difficulties Score, with an inflection point around 25–30 min where the increase accelerated. Similarly, increasing SLP in place of LPA or SED steadily decreased the Total Difficulties Score (more pronounced after 30 min when replacing LPA). Swapping SLP and SED showed symmetric effects, with each 5-minute exchange changing the score by approximately 0.04 in opposite directions.
2. Internalizing Problems (Fig. 2a and b): The dose-response pattern was similar. Increasing MVPA at the expense of LPA or SED continuously decreased Internalizing Problems scores (largest benefit when replacing LPA), while reducing MVPA led to rising scores beyond 25–30 min. Replacing LPA or SED with SLP also decreased Internalizing Problems, with benefits becoming more pronounced after 30 min.
3. Externalizing Problems (Fig. 3): An opposite trend was observed. Increasing LPA (replacing SED or SLP) led to a continuous rise in Externalizing Problems scores (largest increase when replacing SLP). In contrast, increasing SED or SLP (replacing LPA) resulted in decreasing Externalizing Problems, with steeper declines beyond the 25–30 min mark.

These findings underscore the importance of both MVPA and SLP in improving mental health outcomes, particularly beyond the 30 min substitution threshold.

## Discussion

This study used isotemporal substitution and compositional data analysis to examine the relationships between 24 h movement behaviors and preschoolers’ mental health dimensions, including short-term (minute-level) substitutions and dose-response effects of time reallocations. These methods allow a precise interpretation of behavior trade-offs within the fixed 24 h day, which is especially relevant in early childhood. The key findings are as follows: First, the composition of 24 h behaviors was strongly associated with mental health. For example, more MVPA correlated with lower Total Difficulties and Internalizing Problems, whereas more sedentary time was linked to higher Total Difficulties. Greater SLP and SED were associated with lower Externalizing Problems, and no significant associations were observed with Prosocial Behavior. These patterns suggest that different movement behaviors influence specific mental health domains in distinct ways. Second, even brief reallocations of time had notable effects. Substituting as little as one minute of LPA or SED with MVPA or SLP reduced Total Difficulties and Internalizing Problems, highlighting that micro-adjustments in daily routines can benefit mental health. This is especially relevant in early childhood, where sustaining long bouts of activity is challenging. Conversely, replacing SED or SLP with LPA increased Externalizing Problems, particularly Conduct Problems and Hyperactivity/Inattention. Lastly, dose-response analysis showed that increasing MVPA or SLP at the expense of LPA or SED consistently improved Total Difficulties and Internalizing Problems, whereas increasing LPA—especially at the expense of SLP—worsened Externalizing Problems. We identified a critical ~ 30-min threshold beyond which these effects became more pronounced. This suggests that the benefits or detriments of time reallocations intensify beyond a certain duration, offering insight into minimum effective doses for interventions. Overall, these findings highlight the importance of both the overall distribution of movement behaviors and brief activity adjustments in promoting mental health among preschoolers.

Several studies have highlighted the benefits of balancing 24-hour movement behaviors for children’s physical and mental health [12–14]. In line with this evidence, countries such as Canada and Australia have established 24-hour movement guidelines for preschoolers [33–35]. These guidelines recommend that children aged 3 to 5 years have at least 180 min of physical activity per day (including ≥ 60 min of MVPA) and 10–13 h of sleep, with minimal sedentary time. Our compositional analysis showed that, on average, children in our study spent about 78.87 min in MVPA, 112.73 min in LPA, 649.32 min in SED, and 599.08 min in SLP per day. Compared to the guidelines, the average MVPA in our sample met the recommended minimum (≥ 60 min/day), indicating most children achieved the suggested physical activity level. However, average sleep (~599 min, just under 10 h) fell short of the 10–13 h recommended, suggesting some children did not get sufficient rest. These findings demonstrate the utility of compositional data analysis for understanding how preschoolers allocate time across movement behaviors. They also emphasize the need to address not just MVPA but also sleep and sedentary behavior to promote overall health in this age group.

Our SDQ-based analysis highlights complex relationships between movement behaviors and mental health. Compositional regression showed that MVPA was associated with lower Total Difficulties scores and fewer Internalizing Problems (particularly fewer Emotional Symptoms), whereas more SED was associated with higher Total Difficulties. These findings reinforce the positive role of MVPA in preschoolers’ mental health and the potential risks of excessive sedentary time [36]. Previous studies similarly found that MVPA enhances emotional regulation, reduces symptoms of anxiety and depression, and improves cognitive function, all of which contribute to better mental health outcomes [37, 38].

In contrast to internalizing outcomes, increased SED and SLP were associated with improvements in Externalizing Problems. Subdimensional analysis indicated that MVPA was beneficial for reducing Conduct Problems, whereas LPA worsened them. However, MVPA was also linked to higher Hyperactivity/Inattention, whereas both SED and SLP were associated with improvements in that aspect of behavior. These results align with evidence that not all sedentary behavior is detrimental [20, 39]. Non-screen sedentary activities (e.g., reading, structured learning) engage cognitive processes and promote attention regulation, which can reduce impulsivity and hyperactivity in young children [40]. Such activities encourage sustained focus and self-regulation and have a calming influence, explaining why increased SED time can help lower Hyperactivity/Inattention. By contrast, the link between high MVPA and elevated Hyperactivity/Inattention may reflect overstimulation from intense physical activity in preschoolers. Short, unstructured bursts of activity typical at this age can heighten restlessness and distractibility. Thus, while MVPA has clear benefits, its effects vary by behavioral domain and should be structured to avoid exacerbating attention difficulties. The finding that MVPA improves Conduct Problems but worsens Hyperactivity/Inattention underscores the need to tailor physical activity interventions to specific externalizing behavior profiles in preschool children.

To further clarify the impact of time reallocations, we conducted isotemporal substitution analyses. Prior studies applied this approach but often used larger time intervals (e.g., 15 min or more) [20]. Given the rapid, fluctuating nature of preschoolers’ activities, we focused on shorter intervals. We modeled time reallocations in 1- and 5-min increments. The results showed that shifting even 1 min from SED or LPA to MVPA or SLP significantly improved Total Difficulties and Internalizing scores. Conversely, shifting time from SED or SLP to LPA by as little as 1 min increased Externalizing Problems, particularly Conduct Problems and Hyperactivity/Inattention. These effects were evident across all tested intervals, with larger time substitutions producing more pronounced changes.

The finding that even brief changes in activity can significantly affect mental health underscores how sensitive preschoolers’ well-being is to daily activity patterns. Small increases in MVPA or SLP likely yield cumulative benefits for emotional regulation and stress reduction, improving Total Difficulties and Internalizing outcomes [41, 42]. By contrast, replacing SED (especially structured quiet activities) or SLP with additional LPA may overstimulate children. LPA in this age group is typically low-intensity and unstructured, offering less cognitive engagement and fewer opportunities to practice self-regulation [43]. Thus, even modest shifts toward more LPA at the expense of rest or quiet time can increase impulsivity and hinder behavior control, exacerbating externalizing issues [44]. Each small change may seem trivial on its own, but their effects accumulate, highlighting the importance of balancing active play with sufficient rest and calm activities to support optimal mental health.

Our dose-response analysis identified approximately 30 min as a critical threshold where the impact of activity reallocations became markedly stronger. Beyond 30 min, replacing MVPA with LPA or SED more sharply worsened Total Difficulties and Internalizing Problems, and adding SLP in place of LPA led to accelerated improvements in those outcomes. Similarly, substituting LPA with SED or SLP beyond 30 min produced larger gains in Externalizing Problems. These patterns suggest that sustained changes (over 30 min) have cumulative physiological and psychological effects. For example, losing more than 30 min of MVPA may greatly reduce the endorphin release and neurophysiological stimulation needed to manage internalizing symptoms [45]. Such a reduction could significantly diminish these benefits, leading to higher Total Difficulties and more Internalizing Problems.

Conversely, an extra 30 + minutes of sleep allows children to enter deeper, more restorative sleep stages, bolstering emotional resilience and cognitive function [46]. Extended sedentary or sleep periods also help regulate arousal and reduce overstimulation, which can decrease impulsivity and hyperactivity. Notably, longer sedentary periods that involve cognitively engaging, non-screen activities provide opportunities to develop attention span and self-regulation skills. Thus, the 30-minute mark appears pivotal: it illustrates that both the type of activity and its duration influence mental health outcomes. Paying attention to these thresholds is important when designing interventions to optimize preschool children’s psychological well-being.

Moreover, socio-educational factors can introduce additional variation in these associations. For instance, we controlled for parental education level and attitudes toward physical activity, recognizing that higher educational attainment might promote structured routines, while certain cultural or socioeconomic contexts can either limit or facilitate children’s access to safe play environments, consistent sleep schedules, and cognitively stimulating sedentary activities [47]. In homes where parents place a high value on guided reading or quiet play, SED may lean more toward cognitively engaging tasks, potentially buffering against hyperactivity. Likewise, families with rigorous work schedules might struggle to ensure adequate sleep duration and high-intensity physical activities for their children, shaping 24-hour behavior patterns that differentially affect mental health [48]. Though we accounted for these variables in our models, further research could delve into more nuanced aspects of parental involvement, family structure, and broader socio-economic indicators to clarify how these contextual factors interact with 24-hour movement behaviors to influence preschoolers’ mental health.

This study has several strengths. We controlled for a range of covariates—age, gender, residential district, BMI, parental education, and parental attitudes toward physical activity—to reduce confounding and enhance the validity of our findings. We also applied compositional data analysis combined with isotemporal substitution, providing a nuanced understanding of the interdependent relationships between 24 h movement behaviors and mental health. However, certain limitations should be noted. First, the cross-sectional design precludes causal inferences; longitudinal studies are needed to establish temporal relationships. Second, the inference regarding reallocations among 24 h movement behaviors is based on statistical modeling rather than real-life behavioral interventions. Therefore, the observed associations may not fully translate into actual behavioral changes or mental health improvements. Third, although we controlled for socio-educational factors of parents, we did not directly account for child-level factors such as physical disabilities, developmental delays, or existing mental health conditions, which could influence both movement behaviors and psychological outcomes. These unmeasured confounders may limit the generalizability of our findings. Future longitudinal and intervention studies should comprehensively consider both parental and child-related characteristics to validate and expand upon these results. Fourth, we did not differentiate types of sedentary behavior (e.g., screen time vs. non-screen activities), which might have distinct effects on mental health. Future research should examine these qualitative aspects of sedentary time. Fifth, our sample was limited to preschool children in Shanghai, China, which may limit generalizability to other populations and cultural contexts. Studies involving diverse geographic regions and cultural backgrounds are needed to confirm the broader applicability of these findings.

## Conclusion

This study demonstrates that increased MVPA and SLP significantly improve preschool children’s mental health, reducing Total Difficulties and Internalizing Problems. Increased SED, while associated with higher Total Difficulties, showed beneficial effects on Externalizing Problems, particularly in reducing Hyperactivity/Inattention. Even minimal reallocations of time—as little as one minute—from LPA or SED to MVPA or SLP result in notable improvements in mental health outcomes. In contrast, increasing LPA at the expense of SLP or SED exacerbates Externalizing Problems, particularly Conduct Problems and Hyperactivity/Inattention. A critical 30-minute threshold was identified, beyond which the effects of time reallocations on mental health outcomes intensified. In practice, educators, parents, and healthcare providers could schedule short active bouts or restful breaks around this threshold to optimize emotional and behavioral well-being, highlighting the importance of a balanced 24-hour movement approach for preschool children’s mental health.

## Electronic supplementary material

Below is the link to the electronic supplementary material.

Supplementary Material 1.

## Data Availability

No datasets were generated or analysed during the current study.

## Abbreviations

MVPA: Moderate-to-vigorous physical activity
LPA: Light physical activity
SED: Sedentary behavior
SLP: Sleep
SDQ: Strengths and difficulties questionnaire
MPA: Moderate physical activity
VPA: Vigorous physical activity

## References

[1] Barker MM, Beresford B, Bland M, Fraser LK. Prevalence and incidence of anxiety and depression among children, adolescents, and young adults with Life-Limiting conditions: A systematic review and Meta-analysis. JAMA Pediatr. 2019;173(9):835–44. 31282938 10.1001/jamapediatrics.2019.1712 PMC6618774

[2] Polanczyk GV, Salum GA, Sugaya LS, Caye A, Rohde LA. Annual research review: A meta-analysis of the worldwide prevalence of mental disorders in children and adolescents. J Child Psychol Psychiatry Allied Discip. 2015;56(3):345–65. 10.1111/jcpp.12381 25649325

[3] Chai J, Xu H, An N, Zhang P, Liu F, He S, Hu N, et al. The prevalence of mental problems for Chinese children and adolescents during COVID-19 in China: A systematic review and Meta-Analysis. Front Pead. 2021;9:661796. 10.3389/fped.2021.661796 PMC8527981 34692601

[4] Peyre H, Galera C, van der Waerden J, Hoertel N, Bernard JY, Melchior M, Ramus F. Relationship between early Language skills and the development of inattention/hyperactivity symptoms during the preschool period: results of the EDEN mother-child cohort. BMC Psychiatry. 2016;16(1):380. 27821161 10.1186/s12888-016-1091-3 PMC5100106

[5] Shirama A, Stickley A, Kamio Y, Nakai A, Takahashi H, Saito A, Haraguchi H, et al. Emotional and behavioral problems in Japanese preschool children with motor coordination difficulties: the role of autistic traits. Eur Child Adolesc Psychiatry. 2022;31(6):979–90. 33566188 10.1007/s00787-021-01732-7

[6] Agnafors S, Barmark M, Sydsjö G. Mental health and academic performance: a study on selection and causation effects from childhood to early adulthood. Soc Psychiatry Psychiatr Epidemiol. 2021;56(5):857–66. 32813024 10.1007/s00127-020-01934-5 PMC8068628

[7] Li H, Liu K, Fei J, Yuan T, Mei S. Association of early parent-child separation with depression, social and academic performance in adolescence and early adulthood: a prospective cohort study. Child Adolesc Psychiatry Mental Health. 2024;18(1):78. 10.1186/s13034-024-00769-1 PMC11210141 38926788

[8] Wang L, Tian J, Rozelle S. Parenting style and child mental health at preschool age: evidence from rural China. BMC Psychiatry. 2024;24(1):314. 38658866 10.1186/s12888-024-05707-1 PMC11044564

[9] Wlodarczyk O, Pawils S, Metzner F, Kriston L, Klasen F, Ravens-Sieberer U. Risk and protective factors for mental health problems in preschool-aged children: cross-sectional results of the BELLA preschool study. Child Adolesc Psychiatry Mental Health. 2017;11:12. 10.1186/s13034-017-0149-4 PMC5341413 28286550

[10] Brown HE, Pearson N, Braithwaite RE, Brown WJ, Biddle SJ. Physical activity interventions and depression in children and adolescents: a systematic review and meta-analysis. Sports Med (Auckland NZ). 2013;43(3):195–206. 10.1007/s40279-012-0015-8 23329611

[11] Wang W, Zhu Y, Yu H, Wu C, Li T, Ji C, Jiang Y, et al. The impact of sleep quality on emotion regulation difficulties in adolescents: a chained mediation model involving daytime dysfunction, social exclusion, and self-control. BMC Public Health. 2024;24(1):1862. 38992632 10.1186/s12889-024-19400-1 PMC11241850

[12] Carson V, Lee EY, Hewitt L, Jennings C, Hunter S, Kuzik N, Stearns JA, et al. Systematic review of the relationships between physical activity and health indicators in the early years (0–4 years). BMC Public Health. 2017;17(Suppl 5):854. 29219090 10.1186/s12889-017-4860-0 PMC5753397

[13] Poitras VJ, Gray CE, Janssen X, Aubert S, Carson V, Faulkner G, Goldfield GS, et al. Systematic review of the relationships between sedentary behaviour and health indicators in the early years (0–4 years). BMC Public Health. 2017;17(Suppl 5):868. 29219092 10.1186/s12889-017-4849-8 PMC5773886

[14] Chaput JP, Gray CE, Poitras VJ, Carson V, Gruber R, Birken CS, MacLean JE, et al. Systematic review of the relationships between sleep duration and health indicators in the early years (0–4 years). BMC Public Health. 2017;17(Suppl 5):855. 29219078 10.1186/s12889-017-4850-2 PMC5773910

[15] Mekary RA, Willett WC, Hu FB, Ding EL. Isotemporal substitution paradigm for physical activity epidemiology and weight change. Am J Epidemiol. 2009;170(4):519–27. 19584129 10.1093/aje/kwp163 PMC2733862

[16] Chastin SF, Palarea-Albaladejo J, Dontje ML, Skelton DA. Combined effects of time spent in physical activity, sedentary behaviors and sleep on obesity and Cardio-Metabolic health markers: A novel compositional data analysis approach. PLoS ONE. 2015;10(10):e0139984. 26461112 10.1371/journal.pone.0139984 PMC4604082

[17] Dumuid D, Pedišić Ž, Palarea-Albaladejo J, Martín-Fernández JA, Hron K, Olds T. Compositional data analysis in Time-Use epidemiology: what, why, how. Int J Environ Res Public Health. 2020;17(7). 10.3390/ijerph17072220 32224966 10.3390/ijerph17072220 PMC7177981

[18] Dumuid D, Stanford TE, Martin-Fernández JA, Pedišić Ž, Maher CA, Lewis LK, Hron K, et al. Compositional data analysis for physical activity, sedentary time and sleep research. Stat Methods Med Res. 2018;27(12):3726–38. 28555522 10.1177/0962280217710835

[19] Lemos L, Clark C, Brand C, Pessoa ML, Gaya A, Mota J, Duncan M, et al. 24-hour movement behaviors and fitness in preschoolers: A compositional and isotemporal reallocation analysis. Scand J Med Sci Sports. 2021;31(6):1371–9. 33599022 10.1111/sms.13938

[20] Li F, Yin L, Luo W, Gao Z, Ryu S, Sun M, Liu P, et al. Isotemporal substitution effect of 24-hour movement behavior on the mental health of Chinese preschool children. Front Public Health. 2024;12:1288262. 38560447 10.3389/fpubh.2024.1288262 PMC10979542

[21] Ma XK, Zhu Z, Sun C, Zhao S, Cao Z. Characteristics of moderate-to-vigorous physical activity bouts and their relationship with physical fitness in children and adolescents. Sports Sci. 2022;42(4):43–9.

[22] Anderson CB, Hagströmer M, Yngve A. Validation of the PDPAR as an adolescent diary: effect of accelerometer cut points. Med Sci Sports Exerc. 2005;37(7):1224–30. 16015142 10.1249/01.mss.0000170073.57440.df

[23] Pate RR, Almeida MJ, McIver KL, Pfeiffer KA, Dowda M. Validation and calibration of an accelerometer in preschool children. Obes (Silver Spring Md). 2006;14(11):2000–6. 10.1038/oby.2006.234 17135617

[24] Sadeh A, Sharkey KM, Carskadon MA. Activity-based sleep-wake identification: an empirical test of methodological issues. Sleep. 1994;17(3):201–7. 7939118 10.1093/sleep/17.3.201

[25] Meredith-Jones K, Williams S, Galland B, Kennedy G, Taylor R. 24 h accelerometry: impact of sleep-screening methods on estimates of sedentary behaviour and physical activity while awake. J Sports Sci. 2016;34(7):679–85. 26194337 10.1080/02640414.2015.1068438

[26] Stone LL, Janssens JM, Vermulst AA, Van Der Maten M, Engels RC, Otten R. The strengths and difficulties questionnaire: psychometric properties of the parent and teacher version in children aged 4–7. BMC Psychol. 2015;3(1):4. 25815194 10.1186/s40359-015-0061-8 PMC4364334

[27] Mieloo C, Raat H, van Oort F, Bevaart F, Vogel I, Donker M, Jansen W. Validity and reliability of the strengths and difficulties questionnaire in 5–6 year olds: differences by gender or by parental education? PLoS ONE. 2012;7(5):e36805. 22629332 10.1371/journal.pone.0036805 PMC3356337

[28] Yan L, Zhu X, Zhang L, Li Y, Ma Q, Liu P, Chang R, Wang Q, Liu J. Applicability analysis of the strengths and difficulties questionnaire (Parent Version) in 3-7-year-old children with hearing impairment. Chin J Rehabil Theory Pract. 2019;25(8):940–5.

[29] Aarø LE, Davids EL, Mathews C, Wubs AG, Smith ORF, de Vries PJ. Internalizing problems, externalizing problems, and prosocial behavior - three dimensions of the strengths and difficulties questionnaire (SDQ): A study among South African adolescents. Scand J Psychol. 2022;63(4):415–25. 35388463 10.1111/sjop.12815

[30] Chen ST, Liu Y, Tremblay MS, Hong JT, Tang Y, Cao ZB, Zhuang J, et al. Meeting 24-h movement guidelines: prevalence, correlates, and the relationships with overweight and obesity among Chinese children and adolescents. J Sport Health Sci. 2021;10(3):349–59. 32679341 10.1016/j.jshs.2020.07.002 PMC8167320

[31] Dumuid D, Pedišić Ž, Stanford TE, Martín-Fernández JA, Hron K, Maher CA, Lewis LK, et al. The compositional isotemporal substitution model: A method for estimating changes in a health outcome for reallocation of time between sleep, physical activity and sedentary behaviour. Stat Methods Med Res. 2019;28(3):846–57. 29157152 10.1177/0962280217737805

[32] Dumuid D, Stanford TE, Pedišić Ž, Maher C, Lewis LK, Martín-Fernández JA, Katzmarzyk PT, et al. Adiposity and the isotemporal substitution of physical activity, sedentary time and sleep among school-aged children: a compositional data analysis approach. BMC Public Health. 2018;18(1):311. 29499689 10.1186/s12889-018-5207-1 PMC5834855

[33] Chaput JP, Colley RC, Aubert S, Carson V, Janssen I, Roberts KC, Tremblay MS. Proportion of preschool-aged children meeting the Canadian 24-Hour movement guidelines and associations with adiposity: results from the Canadian health measures survey. BMC Public Health. 2017;17(Suppl 5):829. 29219075 10.1186/s12889-017-4854-y PMC5773883

[34] Tremblay MS, Chaput JP, Adamo KB, Aubert S, Barnes JD, Choquette L, Duggan M, et al. Canadian 24-Hour movement guidelines for the early years (0–4 years): an integration of physical activity, sedentary behaviour, and sleep. BMC Public Health. 2017;17(Suppl 5):874. 29219102 10.1186/s12889-017-4859-6 PMC5773896

[35] Okely AD, Ghersi D, Hesketh KD, Santos R, Loughran SP, Cliff DP, Shilton T, et al. A collaborative approach to adopting/adapting guidelines - The Australian 24-Hour movement guidelines for the early years (Birth to 5 years): an integration of physical activity, sedentary behavior, and sleep. BMC Public Health. 2017;17(Suppl 5):869. 29219094 10.1186/s12889-017-4867-6 PMC5773882

[36] Klein AM, Otto Y, Fuchs S, Reibiger I, von Klitzing K. A prospective study of behavioral and emotional symptoms in preschoolers. Eur Child Adolesc Psychiatry. 2015;24(3):291–9. 24972693 10.1007/s00787-014-0575-2

[37] Schuch FB, Bulzing RA, Meyer J, Vancampfort D, Firth J, Stubbs B, Grabovac I, et al. Associations of moderate to vigorous physical activity and sedentary behavior with depressive and anxiety symptoms in self-isolating people during the COVID-19 pandemic: A cross-sectional survey in Brazil. Psychiatry Res. 2020;292:113339. 32745795 10.1016/j.psychres.2020.113339 PMC7384423

[38] Huang Y, Li L, Gan Y, Wang C, Jiang H, Cao S, Lu Z. Sedentary behaviors and risk of depression: a meta-analysis of prospective studies. Translational Psychiatry. 2020;10(1):26. 32066686 10.1038/s41398-020-0715-z PMC7026102

[39] Oh C, Carducci B, Vaivada T, Bhutta ZA. Interventions to promote physical activity and healthy digital media use in children and adolescents: A systematic review. Pediatrics. 2022;149(Suppl 5). 10.1542/peds.2021-053852 10.1542/peds.2021-053852I 35503334

[40] Syväoja HJ, Tammelin TH, Ahonen T, Kankaanpää A, Kantomaa MT. The associations of objectively measured physical activity and sedentary time with cognitive functions in school-aged children. PLoS ONE. 2014;9(7):e103559. 25061820 10.1371/journal.pone.0103559 PMC4111611

[41] Ruiz RM, Sommer EC, Tracy D, Banda JA, Economos CD, JaKa MM, Evenson KR, et al. Novel patterns of physical activity in a large sample of preschool-aged children. BMC Public Health. 2018;18(1):242. 29439704 10.1186/s12889-018-5135-0 PMC5812042

[42] Liu J, Ji X, Pitt S, Wang G, Rovit E, Lipman T, Jiang F. Childhood sleep: physical, cognitive, and behavioral consequences and implications. World J Pediatrics: WJP. 2024;20(2):122–32. 36418660 10.1007/s12519-022-00647-w PMC9685105

[43] Wang J, Wu S, Chen X, Xu B, Wang J, Yang Y, Ruan W, et al. Impact of awareness of sports policies, school, family, and community environmental on physical activity and fitness among children and adolescents: a structural equation modeling study. BMC Public Health. 2024;24(1):2298. 39256716 10.1186/s12889-024-19795-x PMC11389504

[44] Mullen SP, Hall PA, Editorial. Physical activity, self-regulation, and executive control across the lifespan. Front Hum Neurosci. 2015;9:614. 10.3389/fnhum.2015.00614 PMC4635401 26594162

[45] Kettle VE, Madigan CD, Coombe A, Graham H, Thomas JJC, Chalkley AE, Daley AJ. Effectiveness of physical activity interventions delivered or prompted by health professionals in primary care settings: systematic review and meta-analysis of randomised controlled trials. BMJ (Clinical Res ed). 2022;376:e068465. 10.1136/bmj-2021-068465 PMC8864760 35197242

[46] Deng Y, Zhang Z, Gui Y, Li W, Rong T, Jiang Y, Zhu Q, et al. Sleep disturbances and emotional and behavioral difficulties among Preschool-Aged children. JAMA Netw Open. 2023;6(12):e2347623. 38095895 10.1001/jamanetworkopen.2023.47623 PMC10722331

[47] Noble KG, Magnuson K, Gennetian LA, Duncan GJ, Yoshikawa H, Fox NA, Halpern-Meekin S. Baby’s First Years: Design of a Randomized Controlled Trial of Poverty Reduction in the United States. Pediatrics. 2021;148(4). 10.1542/peds.2020-049700 10.1542/peds.2020-049702 PMC8487960 34475270

[48] Wilkes JR, Walter AE, Chang AM, Miller SJ, Sebastianelli WJ, Seidenberg PH, Slobounov S. Effects of sleep disturbance on functional and physiological outcomes in collegiate athletes: A scoping review. Sleep Med. 2021;81:8–19. 33621790 10.1016/j.sleep.2021.01.046

