# Supplementary material for: Reallocating 24-hour movement behaviors and its impact on mental health in preschool children: a compositional data and dose-response analysis
Source: Child Adolesc Psychiatry Ment Health. 2025 May 9;19:52. doi: 10.1186/s13034-025-00911-7 (PMC12065309; doi:10.1186/s13034-025-00911-7)
Supplement: Supplementary file 1 — Supplementary Material 1. [file 13034_2025_911_MOESM1_ESM.docx]

Supplementary Table 1. Variance Matrix of 24-hour Movement Data

| **24-hour Movement** | **MVPA** | **LPA** | **SED** | **SLP** |
| --- | --- | --- | --- | --- |
| MVPA | 0.00 | 0.14 | 0.13 | 0.10 |
| LPA | 0.14 | 0.00 | 0.14 | 0.11 |
| SED | 0.13 | 0.14 | 0.00 | 0.04 |
| SLP | 0.10 | 0.11 | 0.04 | 0.00 |

Supplementary Table 2. Changes in Predicted Mental health with Isotemporal Substitution of 24-hour Movement

|  |  | MVPA↑ | LPA↑ | SED↑ | SLP↑ |
| --- | --- | --- | --- | --- | --- |
| ***Total Difficulties Score*** | 1 minutes reallocated |  |  |  |  |
|  | MVPA↓ | — | 0.022 (0.011,0.033)^*^ | 0.018 (0.009,0.027)^*^ | 0.009 (-0.001,0.019) |
|  | LPA↓ | -0.022 (-0.033,-0.011)^*^ | — | -0.004 (-0.011,0.002) | -0.013 (-0.020,-0.006)^*^ |
|  | SED↓ | -0.017 (-0.026,-0.009)^*^ | 0.004 (-0.002,0.010) | — | -0.009 (-0.013,-0.005)^*^ |
|  | SLP↓ | -0.009 (-0.019,0.001) | 0.033 (0.006,0.020)^*^ | 0.009 (0.005,0.013)^*^ | — |
|  | 5 minutes reallocated |  |  |  |  |
|  | MVPA↓ | — | 0.111 (0.054,0.168)^*^ | 0.090 (0.043,0.137)^*^ | 0.046 (-0.005,0.098) |
|  | LPA↓ | -0.109 (-0.164,-0.053)^*^ | — | -0.023 (-0.054,0.008) | -0.067 (-0.101,-0.032)^*^ |
|  | SED↓ | -0.086 (-0.129,-0.042)^*^ | 0.021 (-0.009,0.051) | — | -0.044 (-0.063,-0.025)^*^ |
|  | SLP↓ | -0.042 (-0.009,0.007) | 0.065 (0.032,0.098)^*^ | 0.044 (0.025,0.063)^*^ | — |
| **Internalizing Problems** | 1 minutes reallocated |  |  |  |  |
|  | MVPA↓ | — | 0.01 (0.002,0.018)^*^ | 0.007(0.001,0.013)^*^ | 0.005 (-0.002,0.011) |
|  | LPA↓ | -0.01 (-0.018,-0.002)^*^ | — | -0.003(-0.007,0.001) | -0.006 (-0.010,-0.001)^*^ |
|  | SED↓ | -0.007 (-0.013,-0.001)^*^ | 0.003(-0.001,0.007) | — | -0.003 (-0.005,-0.001)^*^ |
|  | SLP↓ | -0.004 (-0.011,0.002) | 0.006 (0.001,0.01)^*^ | 0.003 (0.001,0.005)^*^ | — |
|  | 5 minutes reallocated |  |  |  |  |
|  | MVPA↓ | — | 0.051 (0.012,0.090)^*^ | 0.037 (0.005,0.069)^*^ | 0.023 (-0.012,0.059) |
|  | LPA↓ | -0.050 (-0.088,-0.012)^*^ | — | -0.015 (-0.037,0.006) | -0.029 (-0.052,-0.005)^*^ |
|  | SED↓ | -0.035 (-0.065,-0.005)^*^ | 0.014 (-0.006,0.035) | — | -0.013 (-0.026,-0.001)^*^ |
|  | SLP↓ | -0.021 (-0.055,0.012) | 0.028 (0.005,0.051)^*^ | 0.013 (0.001,0.026)^*^ | — |
| Emotional Symptoms | 1 minutes reallocated |  |  |  |  |
|  | MVPA↓ | — | 0.007 (0.002,0.012)^*^ | 0.004 (0.001,0.009) ^*^ | 0.002 (-0.003,0.007) |
|  | LPA↓ | -0.007 (-0.012,-0.002)^*^ | — | -0.003 (-0.006,0.001) | -0.005 (-0.008,-0.002)^*^ |
|  | SED↓ | -0.004 (-0.009,-0.001)^*^ | 0.003(0,0.006) | — | -0.002 (-0.004,-0.001)^*^ |
|  | SLP↓ | -0.002 (-0.007,0.003) | 0.005 (0.002,0.008)^*^ | 0.002 (0.001,0.004)^*^ | — |
|  | 5 minutes reallocated |  |  |  |  |
|  | MVPA↓ | — | 0.036 (0.009,0.063)^*^ | 0.037 (0.005,0.069)^*^ | 0.023 (-0.012,0.059) |
|  | LPA↓ | -0.036 (-0.062,-0.010)^*^ | — | -0.015 (-0.037,0.006) | -0.029 (-0.052,-0.005)^*^ |
|  | SED↓ | -0.022 (-0.042,-0.001)^*^ | 0.013 (-0.001,0.027) | — | -0.013 (-0.026,-0.001)^*^ |
|  | SLP↓ | -0.010 (-0.033,0.012) | 0.025 (0.009,0.040)^*^ | 0.013 (0.001,0.026)^*^ | — |
| Peer Relationship Problems | 1 minutes reallocated |  |  |  |  |
|  | MVPA↓ | — | 0.003(-0.002,0.008) | 0.003(-0.001,0.006) | 0.002 (-0.002,0.006) |
|  | LPA↓ | -0.003(-0.007,0.002) | — | 0 (-0.003,0.002) | -0.001(-0.003,0.002) |
|  | SED↓ | -0.003(-0.006,0.001) | 0 (-0.002,0.003) | — | 0 (-0.002,0.001) |
|  | SLP↓ | -0.002 (-0.006,0.002) | 0.001 (-0.002,0.003) | 0(-0.001,0.002) | — |
|  | 5 minutes reallocated |  |  |  |  |
|  | MVPA↓ | — | 0.015 (-0.008,0.038) | 0.014 (-0.005,0.033) | 0.012 (-0.009,0.033) |
|  | LPA↓ | -0.014 (-0.037,0.008) | — | -0.001 (-0.014,0.012) | -0.003 (-0.017,0.011) |
|  | SED↓ | -0.013 (-0.031,0.005) | 0.001 (-0.011,0.013) | — | -0.002 (-0.010,0.006) |
|  | SLP↓ | -0.011 (-0.031,0.009) | 0.003 (-0.011,0.017) | 0.002 (-0.006,0.010) | — |
| **Externalizing Problems** | 1 minutes reallocated |  |  |  |  |
|  | MVPA↓ | — | 0.001 (-0.008,0.009) | -0.004(-0.011,0.003) | -0.006 (-0.014,0.001) |
|  | LPA↓ | -0.001 (-0.009,0.008) | — | -0.005 (-0.01,-0.001)^*^ | -0.007(-0.012,-0.002)^*^ |
|  | SED↓ | 0.004(-0.003,0.011) | 0.005(0.001,0.009)^*^ | — | -0.002 (-0.005,0.001) |
|  | SLP↓ | 0.006(-0.001,0.014) | 0.007 (0.002,0.012)^*^ | 0.002 (-0.001,0.005) | — |
|  | 5 minutes reallocated |  |  |  |  |
|  | MVPA↓ | — | 0.002 (-0.042,0.046) | -0.021 (-0.057,0.015) | -0.033 (-0.072,0.007) |
|  | LPA↓ | -0.005 (-0.048,0.038) | — | -0.024 (-0.048,-0.001)^*^ | -0.036 (-0.063,-0.009)^*^ |
|  | SED↓ | 0.019 (-0.015,0.054) | 0.023 (0.001,0.046)^*^ | — | -0.012 (-0.027,0.003) |
|  | SLP↓ | 0.031 (-0.007,0.069) | 0.035 (0.009,0.061)^*^ | 0.012 (-0.003,0.027) | — |
| Conduct Problems | 1 minutes reallocated |  |  |  |  |
|  | MVPA↓ | — | 0.006 (0.002,0.01)^*^ | 0.003 (0.001,0.006)^*^ | 0.002 (-0.001,0.006) |
|  | LPA↓ | -0.006 (-0.01,-0.002)^*^ | — | -0.003 (-0.005,-0.001)^*^ | -0.004 (-0.006,-0.001)^*^ |
|  | SED↓ | -0.003 (-0.006,-0.001)^*^ | 0.003(0.001,0.005)^*^ | — | -0.001 (-0.002,0.001) |
|  | SLP↓ | -0.002 (-0.006,0.001) | 0.003 (0.001,0.006)^*^ | 0.001 (-0.001,0.002) | — |
|  | 5 minutes reallocated |  |  |  |  |
|  | MVPA↓ | — | 0.030 (0.011,0.049)^*^ | 0.016 (0.001,0.031)^*^ | 0.013 (-0.004,0.030) |
|  | LPA↓ | -0.030 (-0.048,-0.011)^*^ | — | -0.015 (-0.025,-0.005)^*^ | -0.018 (-0.029,-0.006)^*^ |
|  | SED↓ | -0.015 (-0.029,-0.001)^*^ | 0.014 (0.004,0.024)^*^ | — | -0.003 (-0.009,0.003) |
|  | SLP↓ | -0.012 (-0.028,0.004) | 0.017 (0.006,0.028)^*^ | 0.003 (-0.003,0.009) | — |
| Hyperactivity/Inattention | 1 minutes reallocated |  |  |  |  |
|  | MVPA↓ | — | -0.005 (-0.012,0.001) | -0.007 (-0.013,-0.002)^*^ | -0.009 (-0.015,-0.003)^*^ |
|  | LPA↓ | 0.005 (-0.002,0.012) | — | -0.002 (-0.005,0.002) | -0.004(-0.008,0.001) |
|  | SED↓ | 0.007 (0.002,0.012)^*^ | 0.002(-0.002,0.005) | — | -0.002 (-0.004,0.001) |
|  | SLP↓ | 0.009 (0.003,0.015)^*^ | 0.004 (-0.001,0.008) | 0.002 (-0.001,0.004) | — |
|  | 5 minutes reallocated |  |  |  |  |
|  | MVPA↓ | — | -0.028 (-0.062,0.006) | -0.037 (-0.065,-0.008)^*^ | -0.045 (-0.076,-0.014)^*^ |
|  | LPA↓ | 0.025 (-0.008,0.058) | — | -0.009 (-0.028,0.010) | -0.018 (-0.039,0.003) |
|  | SED↓ | 0.034 (0.008,0.061)^*^ | 0.009 (-0.009,0.027) | — | -0.009 (-0.020,0.003) |
|  | SLP↓ | 0.043 (0.014,0.072)^*^ | 0.017 (-0.003,0.038) | 0.009 (-0.003,0.020) | — |
| ***Prosocial*** | 1 minutes reallocated |  |  |  |  |
|  | MVPA↓ |  |  |  |  |
|  | LPA↓ | — | -0.003 (-0.009,0.004) | -0.002 (-0.007,0.003) | 0 (-0.005,0.006) |
|  | SED↓ | 0.003 (-0.004,0.009) | — | 0.001 (-0.003,0.004) | 0.003 (-0.001,0.007) |
|  | SLP↓ | 0.002 (-0.003,0.007) | -0.001 (-0.004,0.003) | — | 0.002 (0.000,0.004) |
|  | 5 minutes reallocated |  |  |  |  |
|  | MVPA↓ | — | -0.012 (-0.045,0.020) | -0.018 (-0.073,0.037) | 0.002 (-0.058,0.063) |
|  | LPA↓ | 0.013 (-0.019,0.044) | — | 0.009 (-0.028,0.045) | 0.029 (-0.011,0.069) |
|  | SED↓ | 0.009 (-0.016,0.034) | -0.004 (-0.021,0.013) | — | 0.020 (-0.002,0.042) |
|  | SLP↓ | -0.002 (-0.029,0.026) | -0.014 (-0.033,0.005) | -0.020 (-0.042,0.002) | — |

Note: Models are adjusted for covariates (age, gender, district of residence, BMI, parental education level, and parental attitudes toward physical activity). Data are presented as predicted values (95% CI). *P < 0.05; ↑ indicates increased time in the activity; ↓ indicates decreased time in the activity.
